# Liver abscesses with pyopericardium: Laparoscopic management in a preterm neonate

**DOI:** 10.4103/0971-9261.70648

**Published:** 2010

**Authors:** Praveen Ravishankaran, G. Rajamani

**Affiliations:** Department of Pediatric Surgery, Coimbatore Medical College Hospital, Coimbatore, India

**Keywords:** Laparoscopic drainage, liver abscesses, pyo-pericardium

## Abstract

We report a 28-day-old neonate presenting with signs of fever, abdominal distension, and refusal to feed. The baby was diagnosed to have multiple liver abscesses which ruptured and a tract lead to the pericardium resulting in a pyo-pericardium. Laparoscopic drainage of the abscess cavities and the pyo-pericardium was performed. An extensive search of the literature revealed that this case is the youngest one to have undergone such simultaneous laparoscopic drainage.

## INTRODUCTION

There can be multiple routes of infection to liver.[1] Direct invasion from adjacent structures, via hepatic artery, portal veins and umblical veins. Lymph channels along with umbilical vessels can also contribute. Major risk factors for hepatic abscess are sepsis, umbilical catheterization, and omphalitis.[2,3] Minor risk factors include necrotizing enterocolitis, abdominal surgery, maternal infections, infant of diabetic mother, gastroschisis requiring surgery, exchange transfusion, VP shunt and asphyxia neonatorum.

## CASE REPORT

A 28-day-old preterm neonate (34 weeks) was admitted with symptoms of refusal to feeds, high fever, distension in the epigastric region and lethargy. The hematological investigations revealed leukocyte count of 28800/dl. An ultrasonography of abdomen revealed the following finding: Multiple abscesses in the right lobe of liver, three abscesses of 31, 15 and 18 mm in segment 4 of left lobe, abscess of 8 mm in segment 2 of left lobe, large subphrenic abscess of 37×19 mm between the segment 4 and the diaphragm, large subphrenic abscess of 40 mm between segment 2 and the anterior abdominal wall and a tract leading from the abscess cavity of the left lobe of liver to the pericardium resulting in pyo-pericardium.

A decision was taken to drain the abscess cavities and if possible the pyopericardium by laparoscopy. Three 5 mm ports were placed, one in the umbilicus for the 5 mm telescope and two 5 mm working ports each along the midclavicular line, in line with the umbilicus. Multiple abscesses were noted as described in the ultrasound findings. The pus in the peritoneal cavity was aspirated. The liver abscesses were punctured and the pus was aspirated with the help of a suction cannula. The large subphrenic abscess of 40 mm between segment 2 and the anterior abdominal wall was punctured and the pus was sucked out. The epigastric swelling immediately decreased in size. Next search was made to find the tract leading from the abscess cavity to the pericardium. Blunt dissection was made between the left lobe of the liver and the diaphragm. The suction cannula proved to be a very useful instrument for blunt dissection as it causes minimal trauma to the tissues and maximum dissection. Finally the tract leading to the pericardium was located and the suction cannula introduced into it to aspirate out the pus. The telescope was introduced into the pericardium and the amazing view of the beating heart was visualized. There was a small quantity of pus in the pericardium which was aspirated. A drain was placed in the peritoneal cavity and the ports were removed. The postoperative period was uneventful. *Staphylococcus aureus* was grown on culture. The baby was given the necessary antibiotics and was discharged after 5 days.

## DISCUSSION

The management of pyogenic hepatic abscess has changed greatly over the last three decades. Traditionally liver abscess has been regarded as a high-morbidity disease, routinely managed by open drainage, with mortality rates between 70% and 80%.[4] Ultrasound imaging and computed tomography (CT) have made early diagnosis possible and allow accurate guidance of percutaneous aspiration or drainage.[5,6] Laparoscopy also is a very good procedure for draining multiple liver abscesses.

Our case is a 28-day-old preterm neonate born at 34 weeks of gestation presenting with multiple liver abscesses ruptured into the peritoneal cavity. There was also a tract leading from the left lobe abscess cavity through the diaphragm into the pericardium resulting in pyopericardium. The baby was in sepsis, and immediate laparoscopic drainage of the liver abscesses and the pyopericardium was performed. In the process of draining the abscess cavities, the pericardium was entered and the beating heart was visualized. The view of the beating heart was truly amazing and thus proved the effectiveness of laparoscopy in reaching difficult locations and affording excellent images for the same.

A thorough search of literature did not find any similar laparoscopic procedure done on a preterm neonate anywhere in the world. Thus this happens to be the first case of a multiple liver abscess with pyopericardium and pyoperitoneum drained laparoscopically in a preterm neonate. The baby recovered well thus proving the effectiveness of laparoscopy in draining such lesions.
